# Autoimmunity Predisposition in Girls With Turner Syndrome

**DOI:** 10.3389/fendo.2019.00511

**Published:** 2019-07-30

**Authors:** Malgorzata Wegiel, Aleksandra Antosz, Joanna Gieburowska, Kamila Szeliga, Magdalena Hankus, Urszula Grzybowska-Chlebowczyk, Sabina Wiecek, Ewa Malecka-Tendera, Aneta Gawlik

**Affiliations:** ^1^Department of Pediatrics and Pediatric Endocrinology, School of Medicine in Katowice, Medical University of Silesia, Katowice, Poland; ^2^Department of Pediatrics, School of Medicine in Katowice, Medical University of Silesia, Katowice, Poland

**Keywords:** Turner syndrome, autoimmunity, Hashimoto disease, coeliac disease, diabetes mellitus, vitiligo, alopecia areata, psoriasis

## Abstract

**Background:** Turner Syndrome is associated with an increased risk of autoimmune diseases, such as autoimmune thyroiditis, coeliac disease, type 1 diabetes mellitus, inflammatory bowel disease, alopecia areata, or vitiligo. The presence of isochromosome iXq and exposure to estradiol may contribute to the development of the autoimmune process. The aim of this study was to determine the prevalence of autoimmune diseases in a group of TS patients and to assess the impact of karyotype and puberty on the development of autoimmune diseases.

**Patients and Methods:** The analysis encompassed clinical and biochemical data of 134 patients treated between 2001 and 2018. All the patients were examined for autoimmune disease symptoms and tested for the presence of antithyroperoxidase (anti-TPO) and antithyreoglobulin (anti-TG) antibodies. In 73 of the patients, anti-transglutaminase (anti-tTG) antibodies were measured. Thyroid function was assessed by measuring TSH and fT4 levels.

**Results:** The mean follow-up was 5.7 ± 3 years. An autoimmune disease was diagnosed in 46 (34.3%) patients: 39 (29.1%) had only one disorder, whilst 7 (5.2%) presented two disorders. The most common disorder, observed in 40 (29.9%) patients, was thyroid autoimmunity. Hashimoto disease was diagnosed in 20 (14.9%) patients. Of the 73 patients tested for coeliac disease, 4 (5.5%) had anti-tTG and 2 (2.7%) presented overt coeliac disease. Vitiligo was diagnosed in 3 (2.2%) patients, type 1 diabetes mellitus or psoriasis were diagnosed in 2 (1.5%) patients, whilst alopecia areata or lichen sclerosus were diagnosed in 1 (0.7%) patient. The impact of karyotype or estradiol exposure on developing autoimmune diseases were not statistically significant.

**Conclusions:** Our study showed a higher incidence of autoimmune diseases in TS, which is in line with the literature; however, the impact of iXq, or spontaneous/inducted puberty was not confirmed.

## Introduction

One in 2,000–2,500 live born girls is diagnosed with Turner syndrome (TS) caused by a complete or partial absence of one of the X chromosomes (1, 2). Clinical features of this condition include short stature, primary gonadal failure, lymphedema and dysmorphic appearance (3). There is also an increased risk of developing autoimmune diseases, such as: thyroiditis, coeliac disease (CD), diabetes mellitus type 1 (DM1), inflammatory bowel diseases (IBD), alopecia areata (AA), vitiligo (V), psoriasis (P), and lichen sclerosus (LS). The prevalence of autoimmunity increases with age, and more than one autoimmune disease can coexist in one patient (4).

Approximately 40% of TS patients have positive thyroid autoantibodies; however, these patients rarely present any clinical symptoms of thyroid disorders (4–7). Most are diagnosed at the stage of subclinical hypothyroidism (sHT) and approximately a third develop Hashimoto's disease (HD) (5). There are no reports of higher prevalence of Grave's disease in patients with TS (1.7%) (8).

The risk of coeliac disease is 4- to 8-fold higher in TS than in the general population. Screening by endomysial antibodies or tissue transglutaminase antibodies gives positive results in 4.2% of patients and CD is confirmed by biopsy in 85% of screening positive cases (9). The prevalence of IBD in TS patients is 2.6%. Gastrointestinal symptoms often become severe, and in 40% of cases a colectomy is needed (10).

The relative risk of developing psoriasis in TS patients is ~2.25-fold higher than in the general population (11). The prevalence of vitiligo in TS patients is still under debate. Although it is one of the clinical features of TS, its occurrence rate appears similar to the general population (12). Brazzelli et al. report halo nevus as a more common autoimmune skin disorder, with a prevalence of nearly 18% (12). Alopecia areata is reported to affect TS patients 3 times more frequently than the general population (13). Chakhtoura et al. reported lichen sclerosus in 17.3% of their TS patients (14).

Possible factors explaining higher autoimmunity in TS include X-chromosome genes haploinsufficiency, X chromosome origin, excessive production of proinflamatory cytokines, hypogonadism and estrogen therapy (15). Genes *FOXP3* and *PTPN22* are hypothesized to contribute to the pathogenesis. The first one provides instructions for producing the forkhead box P3 (FOXP3) protein—a transcription factor responsible for the development and maintenance of regulatory T cells (Treg) whose function is to prevent the proliferation of effector T cells. The findings in TS patients are inconclusive. Lee et al. demonstrated that although TS patients have a relatively higher number of Treg cells among CD4+ lymphocytes than healthy controls, these cells cannot efficiently suppress an autoimmune reaction (16). The latest study by Gawlik et al. showed that the percentage of Treg cells in girls with TS and coexisting autoimmunity was lower than in the healthy controls and in TS girls without autoimmune diseases (17). Compared with controls, patients with TS also have lower levels of CD4+/CD8+ in peripheral blood (17, 18) as a result of a higher level of CD8+ (18) or a lower level of CD4+ (17). The impact of estrogens and karyotypes on the higher incidence of autoimmunity in TS is under study (17).

The aim of this study was to determine the prevalence of autoimmune diseases in a group of TS girls and to assess the impact of karyotype with isochromosome iXq and puberty on the development of autoimmune diseases.

## Patients and Methods

The data of 134 TS patients treated at the Department of Pediatric Endocrinology between 2001 and 2018 were analyzed. The demographic data of the study population are presented in Table 1.

**Table 1:** Demographic data of 134 girls with Turner syndrome.

	**Mean ± SD [years]**	**Median**	**Min–max**
Age at TS diagnosis [years]	9.0 ± 4.1	9.9	0.0–17.1
Age at 1st examination [years]	9.3 ± 4.1	9.9	0.4–17.1
Follow-up duration [years]	5.7 ± 3.4	5.4	0.0–15.9

*SD, standard deviation*.

TS was diagnosed based on a cytogenetic analysis using peripheral lymphocytes and confirmed by karyotyping with routine G-banding according to the guidelines of the American College of Medical Genetics. All the patients underwent yearly routine visits (from 2 to 4) during which a thorough clinical examination, Tanner staging (19), and blood tests in compliance with the screening recommendations (3) were performed.

The frequency and concomitance of autoimmune diseases were diagnosed based on clinical symptoms and laboratory makers.

P, V, AA, and LS were diagnosed on the basis of medical history as well as skin, scalp, and nail examination during dermatological assessment.

HD was diagnosed based on the presence of antithyroperoxidase antibodies (anti-TPO Ag) or antithyreoglobulin antibodies (anti-TG Ab), or both, and the coexistence of subclinical (isolated elevation of thyroid-stimulating hormone –TSH) or overt (elevated TSH and decreased free thyroxine -fT4) hypothyroidism. TSH and fT4 were measured using chemiluminescent immunometric assay. Concentrations of anti-TPO and anti-TG were determined by enzyme-labeled, chemiluminescent sequential immunometric assay.

Antitissue transglutaminase antibody (anti-tTG) was measured as a screening procedure in all patients diagnosed after 2007 [according to the guidelines (3)] using enzyme-linked immunosorbent assay (ELISA). CD was diagnosed based on clinical and laboratory results (positive serologic markers and duodenal villous atrophy in the biopsy in symptomatic patients or positive serologic markers and tests for HLA DQ2 and DQ8 in asymptomatic patients).

The diagnosis of DM1 was confirmed by hyperglycemia and positive serology (positive antibodies against glutamic acid decarboxylase—GAD, tyrosine phosphatase—IA2, pancreas islets—ICA, and zinc transporter 8—ZnT8).

In the study we analyzed the prevalence of autoimmune diseases depending on karyotype (iXq *vs*. non-iXq patients) and exposure to estrogen: estrogen replacement therapy (ERT) or spontaneous breast development (at least Tanner stage 2).

The study was conducted in accordance with the Declaration of Helsinki. Informed consent was obtained from each patient over the age of 16, a parent or a legal custodian.

## Statistics and Data Analysis

All statistics were performed with STATISTICA version 13. Data are presented as means and SDs, medians, ranges and percentages. Chi-2 test was used to show statistically significant difference in the frequency of autoimmunity within the different karyotype groups (Yates correction was used to compensate for the small sample size). Groups of patients with or without estradiol exposure were compared using Student's *t*-test for two independent samples. The Pearson's correlation was used to show the relation between age at estradiol exposure and age at AD diagnosis. *P*-values of <0.05 were considered significant.

## Results

The mean time of follow-up is shown in Table 1. Autoimmune diseases were diagnosed in 46/134 (34.3%) patients, of whom 39/134 (29.1%) suffered from one disorder and 7/134 (5.2%) presented two coexisting disorders (at the end of follow-up).

### Thyroid Autoimmunology/TA

Positive thyroid autoantibodies were present in 40/134 (29.9%) patients. Anti-TG were detected in 13/134 (9.7%) patients, positive anti-TPO in 4/134 (3.0%); in 23/134 (17.2%) patients, both types of autoantibodies were detected. The diagnosis of thyroid autoimmunity was made after 3.1 years of follow-up: mean age at autoantibodies detection was 13.0 ± 3.3 years (median 13.6, range 5.3–17.9). Autoimmune thyroid disease (HD) was diagnosed in 20/134 (14.9%) patients (mean age 12.1 ± 4.0, median 12.1 years, range 5.3–17.6). None of the girls presented clinical symptoms of hypothyreosis.

There were no cases of Graves-Basedow disease in our study population.

### Coeliac Disease/CD

During the follow-up, 73 TS patients were tested for anti-tTG, with 4/73 (5.5%) obtaining a positive result. However, only 2/73 (2.7%) were diagnosed with CD (diagnosis made at the age of 5 and 7).One patient with isolated autoimmunity and another with overt coeliac disease also presented anti-thyroid antibodies with no clinical symptoms of thyroid disease.

### Type 1 Diabetes Mellitus/DM1

Two patients (2/134, 1.5%) were diagnosed with DM1, one at the age of 2 years old and the other at the age of 11 years old. The first patient also developed thyroid autoimmunity during the follow-up (at the age of 17.7 years).

### Psoriasis/P, Vitiligo/V, Alopecia Areata/AA, Lichen Sclerosus/LS

Out of 134 TS patients, 2 (1.5%) were diagnosed with psoriasis detected at 5.9 and 16.9 years of age, 3 (2.2%) presented with vitiligo diagnosed at the age of 14.4, 11.7, and 12.7 years, 1 (0.7%) presented with lichen sclerosus diagnosed by a pediatric gynecologist at 8 years of age and 1 (0.7%) presented symptoms of alopecia areata at the age of 15.9 years. Positive anti-thyroid autoantibodies were detected in all girls with vitiligo and alopecia, and in one with psoriasis.

### Autoimmunity and Karyotype

There were no statistically significant differences in the frequency or number of autoimmune disorders between groups with isochromosome iXq and with other karyotypes (Table 2).

**Table 2:** Frequency or number of autoimmune disorders between groups with isochromosome iXq and with other karyotypes.

**Karyotype (number)**	**Number (%) of patients with an autoimmune disorder**	**Number (%) of patients with two autoimmune disorders**
iXq (23)	9 (39.1)	3 (13.0)
Non-iXq (111)	37 (33.3)	4 (3.6)
*P*	>0.05	>0.05

*iXq, iso-X-chromosome; non-iXq, other karyotypes*.

*Statistical method: Chi-2 test with Yates correction. Level of significance: P < 0.05*.

### Influence of Estrogens

**Figure 1:**
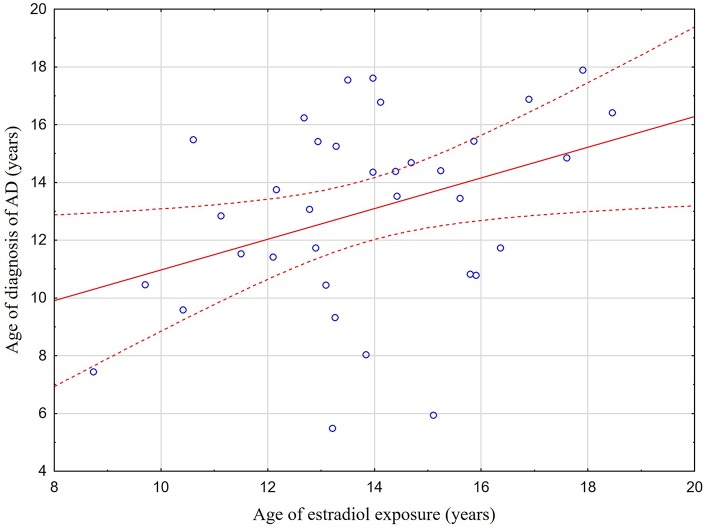
Correlation between the age at estradiol exposure and the age at AD diagnosis. The scatterplot showing the weak positive correlation between the age at estradiol exposure and the age at AD (r = 0.37; *p* = 0.029). Statistical method: the Pearson's correlation. Level of significance: *P* < 0.05.

Out of 46 patients with AD diagnosed during the follow-up, 32 (69.6%) were detected before and 14 (30.4%) after estradiol exposure. The mean age ± SD at positive diagnosis of an autoimmune condition in the two groups was 11.0 ± 4.0 (median 11.6; range 2.0–16.9) and 14.9 ± 2.3 (median 15.3; range 10.5–17.9), respectively. In 36 patients with AD, estradiol exposure started during the follow-up. Figure 1 shows a weak positive correlation between the age at estradiol exposure and the age at AD diagnosis (r = 0.37; *p* = 0.029). There were no statistically significant differences in the age at AD diagnosis between patients with and without estradiol exposure (*p* > 0.05). Table 3 shows which diseases appeared before and which after estradiol exposure.

**Table 3:** Occurrence of autoimmune diseases depending on estradiol exposure.

**AD (number of cases)**	**Number of cases (%) detected before estradiol exposure**	**Number of cases (%) detected after estradiol exposure**
All AD (46)^*^	32 (69.6)	14 (30.4)
TA/HD (40)^**^	27 (67.5)	13 (32.5)
anti-tTG/CD (4)	2 (50)	2 (50)
DM (2)	2 (100)	0 (0)
V (3)	2 (66.7)	1 (33.3)
P (2)	1 (50)	1 (50)
AA (1)	0 (0)	1 (100)
LS (1)	1 (100)	0 (0)

*AD, autoimmune disease; TA, thyroid autoimmunity; HD, Hashimoto disease; anti-tTG, anti-tissue transglutaminase antibodies; CD, coeliac disease; DM, diabetes mellitus type 1; V, vitiligo; P, psoriasis; AA, alopecia areata; LS, lichen sclerosus*.

* *p = 0.0002*.

** *p = 0.0017*.

*Statistical method: the Z-test for two proportions. Level of significance: P < 0.05*.

All cases of clinically overt CD and DM, and 16/20 (80%) cases of HD were detected before estradiol exposure. The presence of autoantibodies typical to CD but without clinical symptoms was detected after estradiol exposure.

## Discussion

The obtained results show that autoimmune diseases are common in patients with TS. During follow up one third of patients developed at least one autoimmune disorder and 5.2% had two coexisting disorders. The most common was TA, present in more than a fourth of patients. Our findings are in line with other studies (4–7). In consistence with previously published data (9), CD occurred more frequently in study group (2.7%) than in the general pediatric population (0.8%) (20), although the prevalence of the disease may have been affected by the fact that not all participants in this study underwent anti-tTG tests. Dermatological assessment revealed V and P in 2.2 and 1.5% of the studied TS girls. Unlike in the adult TS population (14), AA and LS were diagnosed in only one patient (0.7%) each.

All the studied patients with TA were asymptomatic and no one fulfilled the criteria of overt hypothyroidism. Although the presence of thyroid-specific autoantibodies was the only manifestation of thyroid dysfunction in half of our patients with TA, they may develop an overt disease in the future, as presented in previous studies (21–23). By comparing the phenotypic aspects of HD in TS patients and non-TS population, Aversa et al. showed that the biochemical features of thyroid functions are less severe in TS-patients; this may be caused by a less aggressive autoimmune process as well as the increased vigilance of the physicians taking care of TS-patients, thus leading to earlier diagnosis (24).

All the patients diagnosed with two autoimmune conditions suffered from TA. Vitiligo was the most frequent coexisting disease—every case of vitiligo was associated with the presence of thyroid autoantibodies, and ~7.5% of girls with positive anti-TPO or/and anti-TG had vitiligo. The meta-analysis previously performed in non-Turner populations showed that in 20.8% of cases of vitiligo, the presence of thyroid-specific antibodies should be suspected. Conversely, vitiligo occurs in 2.7–7% of patients with TA (25). In the study group, all cases of alopecia and psoriasis were also associated with TA.

Non-syndromic patients suffering from DM1 are more predisposed to developing TA, with a probability of 10–32% (26). In our small subgroup of TS patients with DM1, one was diagnosed with TA.

Collin et al. reported the presence of anti-tTG (with excluded IgA deficiency) in 4.8% of patients with TA, of whom three-fourths were asymptomatic (27). Our data includes one asymptomatic patient with antibodies typical of CD, and one with overt CD and coexisting TA.

Studies performed in non-TS populations revealed that non-thyroidal autoimmune diseases in HD patients tend to appear more frequently in adults. There is also a different spectrum of coexisting disorders in the older population—while in children the comorbidities include mostly DM1 and CD, adults most commonly present with arthropathies and connective tissue diseases. The prevalence of skin diseases is similar in both groups (28).

We assumed the possible impact of the presence of X-isochromosome on developing autoimmune diseases in TS patients. The tendency to develop autoimmune diseases in studied population remains higher in patients with iXq: 40.1% had at least one disorder and 13.6% had two coexisting disorders compared with 30.6 and 4.5% in the non-iXq subgroup. Nevertheless, statistical significance was not reached, possibly due to the small sample size. In the adult population, anti-thyroid autoantibodies were detected in 83% of women with iXq compared with 33% of women with other karyotypes (29). However, the age range of patients in that study was significantly higher than in our study (16–52 vs. 0.4–17), and autoimmunity is a process that evolves with time (5).

The other possible initiator of the autoimmune process considered in this study was estradiol exposure. The impact of estrogens on immune system cells and on pro-inflammatory cytokine regulation is being investigated as a factor for a higher tendency of developing autoimmune diseases in women (30). In this study, nearly 64% of girls were prepubertal at the time of AD diagnosis. Moreover, all cases of overt DM1, CD, and most of HD occurred before estradiol exposure. However, the weak positive correlation between the age at estradiol exposure and the age at AD diagnosis may suggest that the background of autoimmunity in this group was related to the onset of ovarian function or estradiol replacement therapy.

We are aware of the limitations of our study. One of them could be the small sample size. However, the study was conducted in a pediatric population of TS, which is a rare genetic disorder. Regrettably, we were not able to establish the exact number of patients suffering from CD in the entire study group since the guidelines were published after the onset of this study. The subgroup of patients who were subsequently tested for CD were identified in a screening program which started in 2007 (3); a number of patients ended their follow up prior to the publication of the guidelines. Not all the patients with prepubertal diagnosis of AD had estradiol exposure during the study, hence this could potentially introduce a degree of bias in this analysis.

Our results and conclusions from one center study are based on the observation of 134 young patients Turner syndrome with unified follow-up protocol. They refer to previously published findings. It could be considered a positive value, especially that previously published studies were mostly based on even smaller numbers of patients.

Recent guidelines recommend screening for thyroid dysfunction every year and screening for coeliac disease every 2 years (31, 32). Our findings confirm the high prevalence of the two disorders in TS patients, therefore timely screenings may increase the likelihood of diagnosis before the onset of symptoms, and as such contribute to better healthcare. The study shows a higher incidence of autoimmune diseases in TS, which is in line with the literature, however, the impact of iXq and spontaneous or inducted puberty is not confirmed.

## Data Availability

All datasets generated for this study are included in the manuscript and/or the supplementary files.

## Ethics Statement

The study was conducted in accordance with the Declaration of Helsinki and was approved by the Ethical Committee of the Medical University of Silesia. Written informed consent was obtained from the participants' parents or legal custodians and from all participants aged over 16.

## Author Contributions

AG designed the study, analyzed the database, and wrote the manuscript. MW analyzed the database, and wrote the manuscript. AA, JG, KS, MH, SW, UG-C, and EM-T collaborated in designing the work and analyzed the patient results.

### Conflict of Interest Statement

The authors declare that the research was conducted in the absence of any commercial or financial relationships that could be construed as a potential conflict of interest.
